# A Trustworthy LLM-Assisted Optimization Modeling Framework for Remote Sensing Satellite Downlink Scheduling

**DOI:** 10.3390/s26123844

**Published:** 2026-06-17

**Authors:** Yinghui Zhang, Mao Li, Zheng Lu, Zitao Cai, Jingzhe Shan

**Affiliations:** 1 China Academy of Space Technology, Beijing 100094, China; 2 College of Electronic Science and Technology, National University of Defense Technology, Changsha 410073, China

**Keywords:** remote sensing satellite scheduling, downlink scheduling, large language model, optimization modeling, trustworthy verification

## Abstract

This work studies trustworthy use of large language models for remote sensing satellite downlink scheduling. Rather than accepting a generated optimization model at face value, we organize the workflow into three guarded steps: candidate generation, benchmark-based validation, and fallback exact solving. The core technical component is a global time-slicing validator that converts visibility windows into atomic intervals; so, mutual exclusion at the ground-station side, mutual exclusion at the satellite side, and per-satellite download caps can be checked in a physically faithful manner. Results on a prototype instance indicate that LLM-based modeling can be integrated into a dependable scheduling pipeline when external verification and recovery are built into the loop.

## 1. Introduction

Remote sensing satellites generate data continuously, but data return to the ground is limited by brief contact windows and competition for shared stations. This setting connects recent satellite-scheduling work, LLM-based optimization modeling, and broader foundation-model reliability concerns [1,2,3]. For that reason, downlink scheduling remains a recurring bottleneck in remote-sensing and mission-planning workflows [1,4,5]. At the same time, recent large language models have shown that natural-language problem statements can often be translated into mathematical programs and solver-ready code [2,6,7,8,9]. What is still missing, especially in tightly constrained settings, is a reliable rule for deciding when such generated artifacts are safe to trust.

This difficulty is structural rather than cosmetic. A realistic downlink instance may combine overlapping visibility windows, station contention across multiple satellites, and mission- or storage-level limits on each spacecraft [10,11,12]. If even one of these constraints is omitted, the resulting schedule may appear attractive numerically while remaining operationally impossible. That mismatch makes remote sensing satellite downlink scheduling a useful setting for studying trustworthy LLM-assisted optimization [3,13].

Accordingly, this paper does not propose a new scheduling algorithm. Instead, it develops a guarded system architecture in which an LLM acts as a candidate model generator, while acceptance is controlled by an external validator and a fallback exact solver. The key objective is to prevent fluent but invalid model outputs from being returned as final planning recommendations.

The systems question we address is therefore narrow and practical: when an LLM produces a plausible optimization artifact for a high-constraint downlink problem [2,4], how should the surrounding pipeline decide whether to accept it, reject it, or recover from it? Our answer is built around three contributions.

**Contribution 1: Analyst-facing problem framing.** We formalize a workflow in which a user specifies satellites, stations, contact windows, rate assumptions, and optimization goals in natural language and expects the system to produce both a mathematical formulation and executable Gurobi code.
**Contribution 2: External validation mechanism.** We design a validation-centered pipeline that combines structured answer parsing with an exact benchmark based on global time slicing; so, generated candidates are judged against physical consistency rather than surface fluency.
**Contribution 3: Reliable recovery path.** We add explicit acceptance checks and a fallback exact solver, enabling the system to return a dependable schedule even when the generated candidate is incomplete or wrong.

The intended use case is an analyst or researcher who wants modeling assistance but cannot hand over final decision authority to the generator [1,4]. In this setting, readable reasoning is useful, but it is not enough. The returned formulation and code must remain aligned with domain constraints, and the overall workflow must stay usable even when the LLM fails [2,9,14]. This is why the paper emphasizes trustworthy orchestration rather than raw generation quality.

## 2. Related Work

Research on satellite scheduling spans imaging, communication, and integrated observation–communication tasks [1,4,5]. Within that literature, downlink planning stands out, because the temporal overlap and shared infrastructure produce tightly coupled conflicts. Earlier studies therefore focused on exact optimization, heuristics, and decomposition methods that respect operational constraints rather than merely approximate them [10,11,12].

A first relevant line of work addresses formulation issues in the remote sensing satellite downlink itself. Existing models describe visibility windows, exclusive use of communication resources, and upper bounds on mission-level transmission in linear or mixed-integer form [11,12]. This body of work matters here, because it defines the standard against which a generated model should be judged: a schedule is valuable only if it remains physically executable.

A second line of work studies LLM-based optimization modeling. Systems such as OptiMUS, ORLM, and SIRL suggest that language models can already turn problem descriptions into optimization formulations and runnable code [2,8,9,14]. Their promise is reinforced by broader reasoning advances such as chain-of-thought prompting, least-to-most decomposition, self-consistency, and program-aided generation [15,16,17,18,19,20]. Even so, these methods do not eliminate the risk of missing variables, omitted constraints, or code that looks polished but fails to represent the intended model.

A third, less mature direction is external checking of LLM-generated optimization artifacts. In many practical workflows, trust still comes from fluent explanations or limited spot checks. That standard is weak for aerospace planning, where a single overlooked exclusivity constraint can invalidate an otherwise persuasive answer. Our focus differs from prior LLM-for-OR work in that we treat validation and recovery as first-class system components rather than as afterthoughts.

Taken together, this literature motivates the present framework. Satellite scheduling research clarifies what a correct downlink model must preserve, and LLM-for-optimization research shows that automatic model construction is increasingly practical [3,7]. What remains underexplored is how to connect those two developments through benchmark validation, explicit acceptance checks, and a dependable fallback solver. That gap is the main target of this paper.

To further clarify the positioning of the proposed framework, Table 1 compares it with representative LLM-assisted optimization and satellite scheduling approaches from the perspective of validation, trustworthiness, and recovery. The comparison shows that the main novelty of this work centers on a safeguarded orchestration mechanism that combines LLM-based candidate generation with an external physical validator and an exact fallback path for downlink scheduling.

## 3. Problem Formulation

Let S denote the set of satellites and G denote the set of ground stations. For each satellite–station pair (s,g)∈S×G, the visibility window is denoted by $W_{s,g}=\left[a_{s,g},b_{s,g}\right]$ when communication is possible. The scalar transmission rate is *r*, and each satellite has a total data cap $D_{s}^{\max}$. The objective is to maximize the total downloaded data under ground-station exclusivity, satellite exclusivity, and visibility constraints.

We consider a continuous-time abstraction in which transmission can be interrupted and resumed across multiple windows. At any instant, each satellite can downlink to at most one station, and each station can serve at most one satellite. This abstraction captures the main resource conflicts while remaining general enough to support different orbital instances, station layouts, and traffic profiles. The chosen objective emphasizes throughput, but the same framework could be extended to weighted priorities, fairness penalties, or latency-sensitive mission values.

The main challenge is that natural-language descriptions of such systems often leave important details implicit. For example, an LLM may correctly identify window constraints but omit mutual exclusion, or it may produce code that optimizes a surrogate quantity that does not correspond to physically realizable throughput. Therefore, the formulation itself is only one part of the task; the surrounding system must also determine whether the produced model and result are trustworthy [3,13].

## 4. Method

### 4.1. Overall Framework

**Figure 1:**
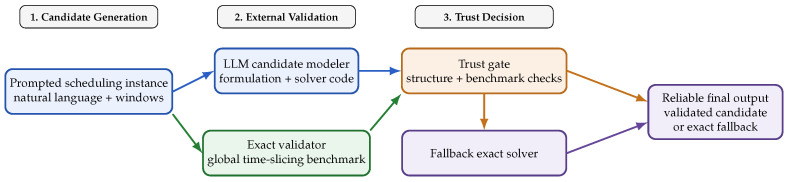
Graphical abstract of the paper: a prompted LLM generates a candidate scheduling model, an external validator checks it against an exact benchmark, and the system returns either the validated candidate or a safe fallback exact solution.

Figure 1 summarizes the full logic of the paper: the system generates a candidate optimization artifact, validates it against an exact benchmark built from the original instance, and returns either the validated candidate or a fallback exact solution.

The LLM first generates a candidate optimization answer with structured sections for reasoning, mathematical modeling, and Python 3.12 code. Then, an exact validator computes a benchmark solution by slicing all visibility boundaries into atomic time intervals. The system finally decides whether to accept the LLM result or switch to the fallback exact solver.

This architecture is intentionally modular. The generation stage is responsible for semantic interpretation of the task description, the validator is responsible for physical consistency, and the fallback solver is responsible for guaranteed recoverability. Such decoupling makes the pipeline easier to audit, because each stage exposes a clear interface and a clear failure mode. In particular, the validator does not need to trust the internal reasoning of the LLM; it only compares extracted quantitative outcomes against a benchmark computed from the original instance.

### 4.2. Prompt Contract and Answer Parsing

To reduce ambiguity at the interface between natural language and optimization code, the system asks the LLM to return a structured answer with four components: a short reasoning summary, a mathematical model, an explicit objective value, and executable Python solver code. This output contract is important because a raw free-form answer is difficult to validate automatically. By requiring explicit fields, the parser can check whether key entities such as decision variables, constraints, and the final objective are all present before the candidate is considered for acceptance [18,19].

The parser is deliberately lightweight. It does not attempt to prove equivalence between the generated model and the intended formulation. Instead, it performs practical checks that are useful in an automated pipeline: whether the objective can be extracted, whether code blocks are present, whether core scheduling entities are referenced, and whether the answer is internally self-consistent. These checks are not sufficient for correctness, but they are effective as a first barrier against malformed or obviously incomplete outputs [15,16,17].

### 4.3. Exact Validator via Global Time Slicing

Let the sorted boundary points of all visibility windows be $\tau_{1}<\tau_{2}<\cdots <\tau_{K}$. We define atomic intervals as

(1) $$I_{k}=\left[\tau_{k},\tau_{k+1}\right],\;k=1,\ldots ,K-1.$$

For each feasible triple (k,s,g), we define a continuous variable $x_{k,s,g}\in \left[0,\Delta_{k}\right]$, where $\Delta_{k}=\tau_{k+1}-\tau_{k}$ and $A_{k}$ denotes the set of satellite–station pairs visible throughout interval $I_{k}$. The objective is

(2) $$\max\sum_{k}\sum_{\left(s,g\right)\in A_{k}}rx_{k,s,g}.$$

The model includes ground-station exclusivity,

(3) $$\sum_{s:\left(s,g\right)\in A_{k}}x_{k,s,g}\leq \Delta_{k},\;\forall k,\forall g,$$

satellite exclusivity,

(4) $$\sum_{g:\left(s,g\right)\in A_{k}}x_{k,s,g}\leq \Delta_{k},\;\forall k,\forall s,$$

and the per-satellite data cap,

(5) $$\sum_{k}\sum_{g:\left(s,g\right)\in A_{k}}rx_{k,s,g}\leq D_{s}^{\max},\;\forall s.$$

Equations (1)–(5) define the interval decomposition, objective, station exclusivity, satellite exclusivity, and data-cap constraint, respectively; each equation covers a distinct part of the validator.

The benefit of this construction is that every feasible schedule can be represented as a collection of decisions on atomic intervals, whose visibility status no longer changes within each interval. This avoids ambiguities that arise when overlapping windows are handled directly at the original window level. It also makes the exclusivity constraints exact: once the boundary set is fixed, each atomic interval behaves like a small continuous resource-allocation block [4,11,12].

In practice, the validator serves two roles. It produces a benchmark objective used to score the LLM output, and it identifies whether the LLM has likely omitted key structural constraints. For example, if a candidate solution exceeds the benchmark by a large margin, this is a strong indicator that some physical restriction has been ignored or incorrectly encoded.

### 4.4. Acceptance and Fallback Policy

The decision module takes as input the extracted objective value, structural sanity checks on the generated answer, and the exact benchmark returned by the validator. A candidate is accepted only when it is syntactically complete, semantically interpretable, and sufficiently close to the benchmark under a chosen tolerance threshold. Otherwise, the system invokes the fallback exact solver and returns its output as the final answer [2,14].

This policy is conservative by design. In aerospace planning, a false positive acceptance is usually more costly than a false rejection, because an infeasible schedule can waste scarce downlink opportunities or create misleading confidence in the automatic modeler. The fallback branch therefore acts as a safety valve: even if generation quality varies across prompts or models, the system still returns a dependable final result.

A simple implementation instantiates the validation score as a normalized objective agreement measure, for example

(6) $$s=1-\min\left(1,\frac{\left|v-b\right|}{b+\epsilon }\right),$$

where *v* is the objective extracted from the candidate, *b* is the benchmark objective, and ϵ>0 avoids division by zero in degenerate cases. Acceptance combines this numerical score with structural checks on the generated answer. This mixed rule reflects a practical lesson from LLM-assisted optimization: numerical closeness is informative when it is accompanied by evidence that the code and formulation are complete.

The threshold is interpreted as an allowable relative objective deviation from the exact benchmark. Equivalently, if δ=1−τ, then a candidate passes the numerical agreement test only when |v−b|/(b+ϵ)≤δ. We use a conservative interpretation of this threshold because the validator computes an independent benchmark for the given instance and because false positive acceptance is more harmful than false rejection in downlink planning. A smaller τ increases the chance of accepting an over-optimistic or structurally incomplete LLM output, whereas a larger τ increases the frequency of fallback calls. Importantly, changing τ affects how often the fallback branch is used, while the exact solver retains feasibility of the final fallback output whenever it is invoked.

### 4.5. Complexity and Scalability

Let *K* denote the number of distinct boundary points induced by all visibility windows. The global time-slicing construction creates at most K−1 atomic intervals, and the number of decision variables scales with the number of feasible interval-link triples (k,s,g). Although this is still polynomial in the discretized representation, the main benefit of the construction is not asymptotic novelty but modeling exactness: it converts overlapping continuous windows into a form that standard linear or mixed-integer optimization tools can reason about consistently.

From a systems perspective, this complexity profile supports prototype-scale validation and characterizes the modeling structure of the global time-slicing validator. The current study establishes how boundary points, atomic intervals, and feasible interval–link triples determine the validator size on the prototype instance. A deployment-oriented scalability assessment can extend this analysis with additional constellation sizes, station networks, visibility-window densities, and data-cap distributions. Reusing the validator across LLMs and prompt templates remains useful, and caching window decompositions, precomputing feasible interval–link sets, and parallelizing candidate evaluation provide practical directions for future deployment-oriented work.

## 5. Experimental Setup

The current prototype uses a six-satellite three-station remote sensing satellite downlink instance with a fixed rate of 0.2GB/min and per-satellite cap of 4.0GB. The benchmark solution is computed by the exact global time slicing validator, following the benchmark-oriented modeling perspective common in satellite scheduling studies [4,5].

**Figure 2:**
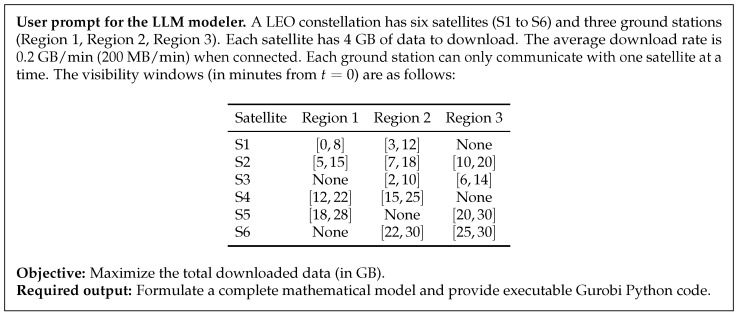
Natural-language prompt used in the prototype downlink-scheduling case study. The prompt specifies the satellite set, ground-station set, visibility windows, per-satellite data cap, transmission rate, objective, and required Gurobi-code output.

The instance is designed to be small enough for transparent analysis, yet rich enough to contain overlapping visibility windows and contention on both the satellite and station sides. The evaluation is organized at the pipeline level, covering benchmark construction, candidate screening, and fallback recovery. Accordingly, the main questions are whether the validator can produce a stable benchmark, whether the acceptance rule can distinguish plausible from unreliable candidate outputs, and whether the fallback mechanism preserves final reliability when the LLM answer is deficient. Figure 2 presents the exact natural-language prompt used to request a mathematical model and executable Gurobi code from the LLM.

Gurobi plays two distinct roles in the prototype pipeline. First, the LLM is asked to produce Gurobi Python code as part of the candidate optimization artifact, and this candidate code is parsed and checked for structural completeness. Second, the exact validator and the fallback exact solver are implemented independently from the LLM output by instantiating the global time-slicing model in Equations (1)–(5). In the current prototype, the independent validator/fallback model produces the benchmark objective, and the LLM-generated code is treated as an artifact for validation. The Gurobi-based exact model provides the external reference for validation and recovery, while fully sandboxed execution of arbitrary generated code forms a future implementation extension.

The reproducibility-relevant workflow is as follows: specify the prompt and visibility windows, construct the global boundary set, instantiate the time-sliced validator from Equations (1)–(5), parse the generated artifact for objective and structural completeness, apply the acceptance policy in Algorithm 1, and invoke the fallback exact solver when any acceptance check fails. The current manuscript documents the workflow, validator construction, and benchmark reference value, while the hardware-dependent runtime measurement is a natural component of future solver-performance benchmarking.

The prototype summary begins with Table 2, which reports the core instance parameters and exact benchmark objective. Table 3 then gives the benchmark allocation summary after the calculation details. The intentionally compact example provides a proof-of-concept demonstration of the pipeline behavior and documents the level of empirical evidence available in the current study.
**Algorithm 1:** Trustworthy LLM-Assisted Downlink Scheduling Pipeline**Require:** Natural-language task description *q*, system prompt *p*, visibility windows *W***Ensure:** Reliable scheduling result *R*  1:Initialize the local LLM generator  2:Generate candidate output o←GENERATE(q,p)  3:Compute exact benchmark b←GLOBALTIMESLICING(W)  4:Extract objective value v←EXTRACTOBJECTIVE(o)  5:Score s←VALIDATE(v,b)  6: **if** s≥τ and *o* is valid **then**  7:    R←o  8:**else**  9:    R←FALLBACKEXACTSOLVE(W)10:**end if**11:**return***R*

**Figure 3:**
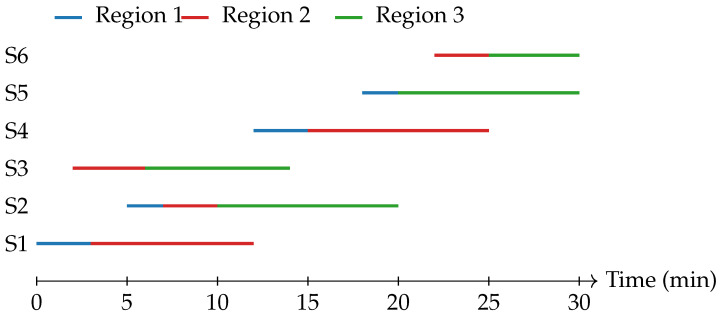
Visibility windows of the six-satellite three-station prototype instance. The line colors in the legend match the line colors used for the corresponding ground-station windows. Different colors represent different ground stations, and overlapping windows create the station-side and satellite-side contention that must be handled by the validator.

In addition to the mechanism-level comparison, we discuss how the system is intended to behave under distinct categories of generation error without reporting unobserved statistical error frequencies. These are realistic failure patterns for prompt-based optimization assistants, because the model may understand the narrative structure of the task while still missing one decisive technical detail. Figure 3 visualizes the overlap structure of the six-satellite instance.

For the prototype instance, the global boundary set is {0,2,3,5,6,7,8,10,12,14,15,18,20,22,25,28,30}, which yields 16 atomic intervals. Solving the time-sliced linear model gives a total scheduled transmission time of 68min. Under the fixed rate of 0.2GB/min, this corresponds to the benchmark objective of 13.6GB. This independently computed benchmark serves as the external reference for validating the LLM candidate.

Table 3 reports the corresponding allocation summary to improve transparency of the benchmark solution.

Table 4 compares three deployment modes: LLM only, LLM plus external validation, and the full pipeline with both validation and fallback.

This comparison provides a mechanism-level account of the current prototype. Since the present study evaluates a single prototype instance, the table reports the safeguards implemented in the pipeline. A larger benchmark-scale evaluation can additionally report quantitative metrics such as the code executability rate, solution feasibility rate, objective agreement rate, and constraint violation rate.

The acceptance signals in Table 5 are designed as operational checks in the prototype pipeline. In the current study, they specify how a candidate artifact is parsed, compared with the benchmark, and routed to the fallback branch. Future benchmark-scale experiments can extend these operational checks with quantitative indicators such as code executability rate, solution feasibility rate, objective agreement rate, and constraint violation rate across multiple instances, prompts, and LLM backbones.

## 6. Results and Discussion

The prototype results support the main system-design claim of the paper: the proposed framework contributes a controlled acceptance, rejection, and fallback-replacement process for LLM-generated scheduling models.

First, the exact benchmark on the prototype instance is 13.6GB, as reported in Table 2. This value reflects the coupled effects of visibility windows, station-side exclusivity, and satellite-side exclusivity. The instance therefore provides a compact scheduling case with genuine resource contention for evaluating the guarded pipeline.

Second, at the mechanism level, Table 4 illustrates how external validation changes the role of the LLM from an unchecked answer generator into a candidate modeler whose output must be compared with an independent benchmark. That shift is central to the trust argument of the paper.

Third, the fallback branch turns rejection into recovery in the prototype workflow. This matters in the analyst-facing setting studied here, because the user ultimately needs a usable result after generation fails. The full pipeline provides a recovery path when generation is incomplete and supports dependable final behavior in the evaluated prototype.

The failure modes summarized in Table 6 further illustrate why a single acceptance heuristic is insufficient. Some bad outputs are numerically suspicious, while others look numerically plausible but remain structurally incomplete. A candidate may report a near-benchmark objective and still omit runnable code or key constraints. Conversely, a syntactically polished answer can still fail under external comparison. These cases motivate the combined use of parsing, quantitative validation, and fallback solving.

Table 6 summarizes representative failure categories in LLM-assisted optimization modeling and connects each category to the corresponding system response. The current proof-of-concept study establishes this failure-handling taxonomy on the prototype instance. Repeated generations across multiple LLMs, prompts, and scheduling instances can further quantify the frequency of these errors in future benchmark-scale studies.

The current study presents a proof-of-concept system prototype with external validation, acceptance gating, and exact fallback recovery on a transparent downlink instance. The implementation currently checks generated solver artifacts through parsing and structural completeness checks, while sandboxed execution and broad synthetic benchmark stress testing form natural extensions of the prototype. Future work should extend the benchmark set, introduce richer task priorities and time-varying rates, and compare multiple open-source LLMs under the same validator.

## 7. Threats to Validity

This study has several clearly defined limitations. First, the experimental evidence is intentionally focused: the prototype evaluates one illustrative downlink instance and uses it to demonstrate the system design, validation workflow, and recovery mechanism. This positioning makes the paper a proof-of-concept system-design study with a transparent benchmark instance and explicit directions for broader empirical comparison across models and scheduling datasets.

With one prototype instance, the present results characterize the validator’s modeling structure and demonstrate the guarded workflow on a transparent scheduling case. The study identifies the instance dimensions that govern broader generalization analysis, including constellation size, station-network size, visibility-window density, and data-cap distribution. A stronger empirical evaluation can build on this foundation through a synthetic or real benchmark suite with multiple instance scales and repeated LLM generations under controlled prompt and solver settings.

Second, the current validation target combines final-objective agreement with structural plausibility checks on the generated answer. Two formulations may share the same objective on a small instance while differing on other cases. For this reason, benchmark agreement functions as a strong practical signal for the prototype workflow and motivates future semantic-equivalence checks across broader instance families.

Third, the human factors dimension of LLM-assisted optimization remains an important extension, including how analysts calibrate trust when explanations appear technically fluent. This issue is especially relevant in aerospace settings, where confidence calibration can be as important as raw optimization quality. Addressing these threats will benefit from larger benchmark suites, execution sandboxes, and user studies.

## 8. Conclusions

This paper presented a trustworthy LLM-assisted optimization modeling framework for remote sensing satellite downlink scheduling. The main idea is straightforward: generated formulations pass through benchmark validation and, when necessary, are replaced by an exact fallback solution.

Under this view, the central question is how to place LLM modeling capability inside a pipeline that continues to behave reliably when the generated model is incomplete, inconsistent, or simply wrong. For high-constraint aerospace planning, such guarded orchestration provides a practical decision-support structure around generated formulations.

The current evidence provides a proof-of-concept demonstration of a guarded modeling workflow with independent validation and fallback recovery. Future work should extend the evaluation to larger benchmark suites, execute generated solver code in sandboxed environments, and report quantitative reliability metrics across multiple LLMs and scheduling scenarios.

## Figures and Tables

**Table 1:** Comparison between representative LLM-assisted optimization frameworks and the proposed trustworthy orchestration framework.

Framework Type	Main Focus	Validation Mechanism	Trustworthiness Feature	Recovery Strategy
General LLM-to-optimization modeling	Generate mathematical formulations and solver code from natural language	Mainly syntax checks, solver feedback, or answer-level consistency checks	Improves modeling automation but may still accept incomplete domain constraints	Usually relies on regeneration, refinement, or user correction
Solver-informed or training-based LLM optimization	Improve model generation through solver signals or optimization-oriented training	Solver interaction or learning-based feedback	Strengthens generation quality, with domain-specific physical validation supplied by an external safeguard when required	Focuses on generation improvement, with fallback scheduling handled by a separate recovery module
Satellite scheduling optimization	Construct exact, heuristic, or decomposition-based scheduling algorithms	Feasibility and optimality are checked inside the scheduling model itself	Strong domain correctness, but not designed for LLM-generated artifacts	Solver or heuristic output is the final result
Proposed framework	Trustworthy orchestration of LLM-generated downlink scheduling models	External global time-slicing validator based on the original visibility windows	Explicit structural checks, benchmark agreement, and physical consistency verification	Fallback exact solver returns a dependable schedule when the LLM candidate is rejected

**Table 2:** Benchmark result on the prototype instance. The objective is computed by the independent global time-slicing validator and is used as the reference value for accepting or rejecting the LLM candidate.

Metric	Value	Note
Satellites	6	S1–S6
Ground stations	3	Region 1, Region 2, Region 3
Rate	0.2GB/min	fixed
Per-satellite limit	4.0GB	total upper bound
Benchmark objective	13.6GB	exact global time slicing

**Table 3:** One benchmark allocation summary obtained by the global time-slicing validator. The objective value is 13.6GB, corresponding to 68min of total scheduled downlink time at 0.2GB/min.

Satellite	Time (min)	Data (GB)	Ground Station	Time (min)	Data (GB)
S1	12	2.4	Region 1	26	5.2
S2	14	2.8	Region 2	28	5.6
S3	12	2.4	Region 3	14	2.8
S4	13	2.6	Total	68	13.6
S5	12	2.4			
S6	5	1.0			

**Table 4:** Mechanism-level comparison of different pipeline settings. The table describes the safeguards available in the current proof-of-concept evaluation.

Setting	Benchmark Comparison	Fallback Available	Main Residual Risk
LLM only	No	No	Fluent but infeasible formulations may be accepted
LLM + validation	Yes	No	Invalid candidates can be rejected, but no final schedule is guaranteed after rejection
LLM + validation + fallback	Yes	Yes	Rejected candidates are replaced by the exact fallback solution

**Table 5:** Acceptance signals used before returning the LLM candidate. These signals combine structural parsing, objective extraction, benchmark agreement, constraint cues, and fallback readiness.

Signal	Purpose	Typical Rejection Trigger
Objective extraction	Obtain comparable scalar output	Missing or ambiguous final objective
Structure parsing	Verify answer completeness	No model, no code, or malformed sections
Benchmark agreement	Compare with exact validator	Large gap between candidate and benchmark
Constraint cues	Check domain entities appear	Missing station or satellite exclusivity
Fallback readiness	Guarantee reliable return value	Candidate rejected after any prior check

**Table 6:** Representative failure modes handled by the proposed pipeline. The listed categories summarize illustrative failure types covered by the current prototype analysis.

Failure Mode	Observable Symptom	System Response
Missed exclusivity	Candidate objective unrealistically exceeds exact benchmark	Reject candidate and call fallback solver
Objective mismatch	Reported value inconsistent with extracted model semantics	Mark output unreliable during parsing
Incomplete code	Missing variables, constraints, or solver call	Fail structural checks before acceptance
Prompt ambiguity	Reasonable text but underspecified optimization details	Use validator as external reference

## Data Availability

The data supporting the findings of this study are not publicly available due to institutional confidentiality requirements and project restrictions of the authors’ affiliation. Requests for data access may be directed to the corresponding author and will be considered subject to institutional approval.
